# The High-Quality Genome Sequencing and Analysis of Red Raspberry (*Rubus idaeus* L.)

**DOI:** 10.1155/2024/9271183

**Published:** 2024-11-25

**Authors:** Haopeng Zhang, Weihua Li, Guodong Li, Jiaren Liu, Hongsheng Chen, Chunpeng Zhang, Jinlu Zhao, Zhicheng Zhang, Qiang Lv, Yan Zhang, Guohui Yang, Ming Liu

**Affiliations:** ^1^Department of Oncological Surgery, The Fourth Affiliated Hospital of Harbin Medical University, Harbin 150001, China; ^2^Department of Medical Imaging, Shenzhen Second People's Hospital/The First Affiliated Hospital of Shenzhen University, Shenzhen 518035, China; ^3^Department of General Surgery & Bio-Bank of General Surgery, The Fourth Affiliated Hospital of Harbin Medical University, Harbin 150001, China; ^4^Department of Clinical Laboratory, The Fourth Affiliated Hospital of Harbin Medical University, Harbin 150001, China; ^5^Computational Biology Research Center, Harbin Institute of Technology, Harbin 150000, China; ^6^College of Horticulture and Landscape, Northeast Agricultural University, Harbin 150030, China

**Keywords:** genome annotation, Hi-C, PacBio SMRT sequencing, *Rubus idaeus* L.

## Abstract

Red raspberry (*Rubus idaeus* L.), which is an important nutritional source for human health, belongs to fruit crops of the Rosaceae family. Here, we used Pacific Biosciences single-molecule real-time (SMRT) sequencing and high-throughput chromosome conformation capture (Hi-C) sequencing technologies to assemble genomes and reported a high-quality *Rubus idaeus* L. (DNS-1) genome with 321.29 Mb assembled into seven chromosomes. The LAI score of the DNS-1 genome assembly was 21.32, belonging to gold quality. Approximately 52.3% of the assembly sequences were annotated as repetitive sequences, and 24.15% were composed of long terminal repeat elements. A total of 29,814 protein-coding genes and 2474 pseudogenes were predicted in DNS-1. We characterized the complete genomes of DNS-1 and compared them to those of seven other species. We found that 652 gene families were unique to DNS-1 and they were shaped from an ancestor. There were 1000 and 5193 gene families that expanded and contracted in the DNS-1 genome. The *Rubus idaeus* L. genome can be used to understand the structure and evolution of Rosaceae genomes and can be developed to identify genes controlling important traits and improve breeding work.

## 1. Introduction

Red raspberry (*Rubus idaeus* L.) has received much more attention for its health benefits, due to the nutritional value and economic value. The fruit of red raspberry can be used not only as a nutritious processed food, such as juice, jelly, and alcoholic beverages, but also as a natural medicine to treat obesity, cardiovascular diseases, and Type 2 diabetes. The earliest cultivated raspberry was brought to Heilongjiang Province as domesticated fruit crops by the Russian emigrants from the coastal region in 1915. The industry of raspberry began in the mid-1980s and developed rapidly in China in recent decades. The raspberry industry was restricted due to lack of breeding technology, information resources, the capacity of deep processing, and scientific research.

Red raspberry is diploid (2*n* = 2*x* = 14) and belongs to fruit crops of the Rosaceae family, which also includes other genome-sequenced plants such as apple (*Malus × domestica*) [1], strawberry (*Fragaria vesca* and *Fragaria × ananassa*) [2, 3], pear (*Pyrus bretschneideri*, *Pyrus communis*, and *Pyrus pyrifolia*) [4–6], peach (*Prunus persica*) [7], and sweet cherry (*Prunus avium*) [8]. The genomes of all species in the Rosaceae family have not been fully deciphered, and there are variations in the genomes due to species, location, and growth patterns. Here, using the Illumina sequencing, PacBio sequencing, and Hi-C sequencing approach, we reported a genome of red raspberry (*Rubus idaeus* L.) selection DNS-1. DNS-1 was carefully cultivated by Northeast Agricultural University, which had a multitude of characteristics, including cold and disease resistance, good overwintering, vigorous plant growth, strong ability to form basal branches, and easy to root tiller. The *Rubus idaeus* L. genome can be used to understand the structure and evolution of Rosaceae genomes and can be developed to identify genes controlling important traits and improve breeding work.

## 2. Materials and Methods

### 2.1. Plant Materials

*Rubus idaeus* L. DNS-1 were selected for sequencing because of their important character resistance to cold and diseases for genetic research. The plants were cultivated by Northeast Agricultural University, and the roots, stems, and leaves from the same plant were gained and frozen by liquid nitrogen for extraction of genomic DNA.

### 2.2. PacBio Sequencing

The genomic DNA was extracted from roots, stems, and leaves of DNS-1 using the CTAB method. To construct and sequence the library, a g-TUBE device was used to shear the genomic DNA. Sheared DNA was repaired and transited by zero-mode waveguides used for single-molecule real-time (SMRT) sequencing on a PacBio Sequel system according to the manufacturer's protocol (Pacific Biosciences). To construct the library, the BluePippin Size Selection System was used to target segment screening with a 20-kb template. The sequencing and library preparation were done at the Biomarker Technologies Corporation (Beijing, China).

### 2.3. Illumina Sequencing

The library was constructed with a 270-bp insert fragment for DNS-1 according to the manufacturer's protocol (Illumina). The genomic DNA was fragmented to the target fragment (270 bp) by ultrasonic shock. Using the PE150 strategy, the libraries were constructed on a HiSeq 2500 system through the steps of (i) terminal repair of target fragment, (ii) adenine addition to the ends of the fragments, (iii) single T addition to the 3⁣′end, and (iv) target fragment selection.

To determine if the extracted DNA was contaminated or not, we selected 10,000 single-ended reads randomly from the sequenced library and blasted them by alignment to the NT database using ncbi-blast+ 2.2.29 with default parameters. Contaminated reads, such as chloroplast and mitochondrial sequences (XM111009, 410,592 bp), were screened by SOAP v2.21 with default parameters. The high-quality reads were used for estimation of genome size and heterozygosity based on *k*-mer frequency (*k* = 19). Finally, we generated a total of 14.94 Gb (~48.70×) clean Illumina reads for DNS-1. The distribution of distinct *k*-mers (*k* = 19) showed peaks at 41 (Figure S1), and 306.79 Mb of genome size was estimated from the higher peak. The results suggested that the heterozygosity of the genome was high.

### 2.4. Hi-C Sequencing Data

According to the Hi-C procedure, Hind III was used as a restriction enzyme to cut the nuclear DNA. The cohesive ends of these fragments were biotinylated by biotin-labeled bases. Cyclization between ligated DNA fragments was performed to determine the location of the interacting DNA.

Biotinylated circle–chimera DNA molecules were sheared to 300–700 bp and sequenced by using an Illumina HiSeq instrument. After read filtering, we obtained 21.93 million valid interaction pairs for assembly of DNS-1. The libraries of Hi-C data were analyzed with the HiC-Pro pipeline [9]. The contigs were divided into fragments with an equal length of 100 kb and were then clustered using LACHESIS software [10] with default parameters (CLUSTER_MIN_RE_SITES = 43, CLUSTER_MAX_LINK_DENSITY = 2, CLUSTER_NONINFORMATIVE_RATIO = 2, ORDER_MIN_N_RES_IN_TRUN = 25, and ORDER_MIN_N_RES_IN_SHREDS = 21). Total lengths were anchored and oriented to their respective chromosome groups. In addition, the assemble evaluation was performed by heat map, Quickmerge software, and Benchmarking Universal Single-Copy Orthologs (BUSCO).

### 2.5. Genome Assembly

The whole-genome assembly was conducted using the Falcon sense method [11] and a Canu-based pipeline [12] with high-quality reads. The result of genome assembly was optimized by Quickmerge software [13]. The clean reads were integrated to correct the InDels and SNPs in the assemblies using Pilon v1.22 [14].

### 2.6. Evaluation of Assembly Quality

To evaluate the completeness and accuracy of the assembly, 458 core eukaryotic genes (CEGs) and 1440 BUSCO were mapped to the genomes using Core Eukaryotic Genes Mapping Approach (CEGMA) v2.5 [15] and BUSCO v2.0 [16].

Long terminal repeat (LTR) Assembly Index (LAI) was used to check for genome assembly quality [17]. The following steps were applied to calculate LAI for a genome assembly. First, we obtained LTR retrotransposon candidates using LTR-FINDER v1.0.5 and LTRharvest v1.5.9. Then, we used LTR_retriever v2.8 with default parameters to retain intact LTR-RTs and filter out false candidates. Finally, we calculated the LAI score. The program was freely available through GitHub: https://github.com/oushujun/LTR_retriever.

### 2.7. Repeated Sequence Annotation

LTR-FINDER v1.0.5 [18], MITE-Hunter [19], RepeatScout v1.0.5 [20], and PILER-DF [9] were employed to estimate the repeat components by building a repeat library, and the results were classified using PASTEClassifier v1.0 [21]. The final repeat library was created combined with the Repbase database [22] and the constructed database. Repeat sequences in *Rubus idaeus* L. were finally identified by RepeatMasker v4.0.6 [23].

### 2.8. MegaLTR

MegaLTR, a web server and standalone pipeline, was used to detect intact LTR-RTs at the DNS-1 genome. Detailed workflow could be found in the available web (https://github.com/MoradMMokhtar/MegaLTR).

### 2.9. MegaSSR

MegaSSR, a robust online server, was used for large-scale simple sequence repeat (SSR) identification, classification, and marker development. Detailed workflow could be found in the available web (https://github.com/MoradMMokhtar/MegaSSR).

### 2.10. Prediction of Protein-Coding Gene and Pseudogenes

Gene structures were annotated using two methods (ab initio predictions and homology-based proteins). Augustus v2.4 [24], GlimmerHMM v3.0.4 [25], Genscan v1.0 [26], GeneID v1.4 [27], and SNAP [28] were used for gene prediction. *Arabidopsis_thaliana*, *Fragaria_vesca*, *Fragaria iinumae*, *Fragaria nipponica*, and *Fragaria nubicola* were used for the prediction of homologous species by GeMoMa v1.3.1 software [29]. Finally, these consensus genes were integrated by EVM v1.1.1 [30] and modified by PASA v2.0.2. The GeneWise v2.4.1 software [31] was used for predicting pseudogenes in the *Rubus idaeus* L. genome.

### 2.11. Gene Annotation

Using the software BLAST v2.2.31 with the parameter “*E*‐value = 1e^−5^,” the sequences of predicted genes were blasted against nucleotide and protein sequence databases, such as NR (https://ftp.ncbi.nlm.nih.gov/blast/db/FASTA/), KOG [32], Gene Ontology (GO) [33], Kyoto Encyclopedia of Genes and Genomes (KEGG) [34], and TrEMBL [35], and the results were annotated.

### 2.12. Gene Family and Phylogenetic Analyses

A total of 279,035 genes from eight plant species were used for clustering analysis of the gene family. Using default settings for the parameters, the OrthoMCL [36] software was used to cluster gene families. Using single-copy protein sequences among the eight species, PhyML [37] software was used to construct a phylogenetic tree with the general time-reversible model. Divergence times for the eight species were performed by the MCMCTree method as implemented in the PAML package (v.4.4) [38]. CAFE (v.2.1) [39] was used to identify the expansion and contraction of gene families, and a functional annotation analysis of expansion gene families was conducted.

### 2.13. Insert Time for LTR and Whole-Genome Duplication (WGD)

We used LTR_FINDER [18] to find LTRs, which could calculate the insert time with the formula time = *K*/2*r*. *K* is the distance between alignment pairs and calculates with the program, which is implemented in the Kimura two-parameter model. *r* is the rate of nucleotide substitution, which is set to 7.3 × 10^−9^. The fourfold degenerate site (4DTv) for each gene pair was calculated for the percentage of transversions at all fourfold degenerate third codon positions. We used the distribution of 4DTv to detect WGD events and estimate homologous gene mutation in the *Rubus idaeus* L. genome.

## 3. Results

### 3.1. Genome Sequencing and Assembly of the Red Raspberry

Using the PacBio SMART platform, we sequenced the *Rubus idaeus* L. genome. Total bases of 26.43 Gb and an average length of 14.37 kb were obtained (shown in Table S1). After assembly using the Canu-based pipeline [12] and Falcon [11], Quickmerge [13], and Pilon software [14], we generated a *Rubus idaeus* L. (DNS-1) genome of 485.03 Mb with a contig N50 of 4.91 Mb and the GC content was 37.91% (shown in Table 1). The accuracy and completeness of the assembly were examined with CEGMA assessment [15] and BUSCO [16]. In 458 CEGs, 440 were completely present, indicating that 96.06% of the CEGs were detected. In 248 highly conserved CEGs, 235 were completely present, indicating that 94.76% of the highly conserved CEGs were detected. The results of assessment showed the high completeness of the assembly (shown in Table S2). Using a benchmark of 1440 conserved plant genes, the BUSCO method was used to assess the completeness of gene regions, and the results revealed that the integrity was 92.50% (complete BUSCO/total BUSCO), while the percentage of duplicated BUSCO was 28.68%, indicating that the result of the assembly contained a large number of redundant sequences (shown in Table S3 and Figure S2).

**Figure 1:**
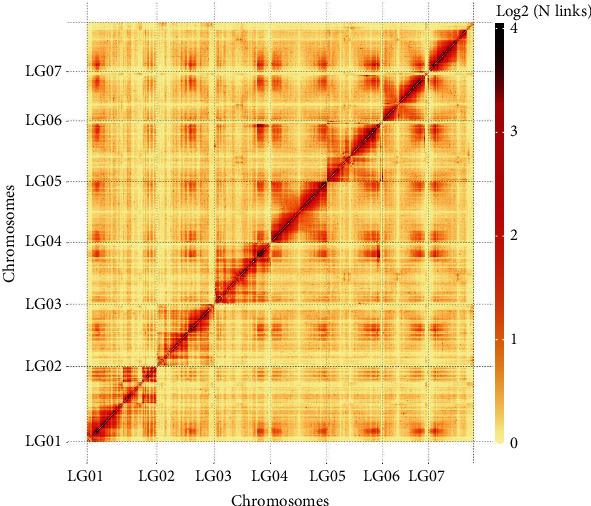
Genome-wide Hi-C map of *Rubus idaeus* L. (DNS-1). Hi-C interaction matrices represent the interaction links of ordered scaffolds. The intensity of light yellow to dark red represents the frequency of Hi-C correlations from low to high on a logarithmic scale.

**Figure 2:**
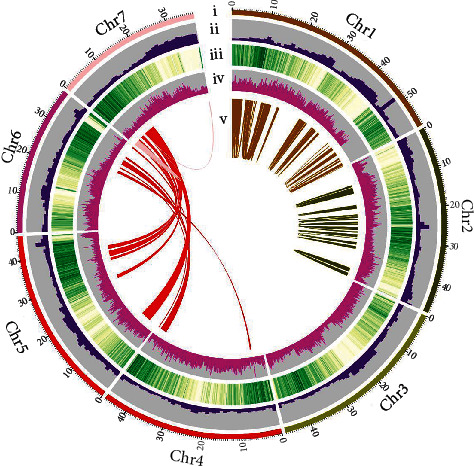
Genome landscape of *Rubus idaeus* L. (DNS-1). Elements indicate tracks from the outer to inner circles: (i) syntenic relationships among chromosomes of *Rubus idaeus* L., (ii) the distribution of GC content in the *Rubus idaeus* L. genome, (iii) gene density, (iv) distribution of repeats, and (v) the internal collinearity of the *Rubus idaeus* L. genome.

The aim of the Hi-C library was used to anchor contigs to their respective chromosome groups. Here, we generated 27.34 Mb unique paired alignments and 21.93 Mb valid Hi-C interaction pairs for the DNS-1 (shown in Table S4). Contig was interrupted by 100 kb in equal length and reassembled by Hi-C. A total of 482.43 Mb of genome sequences were located on chromosomes (shown in Table S5). A Hi-C heat map, in which the genome was divided into bins with equal length and the contacted number of each pair was observed [40], indicated that the genome was correctly assembled (shown in Figure 1 and Figure S3). Hi-C optimized genome was corrected using Pilon software, and the final genome size was 321.29 Mb, with a contig N50 of 2.36 Mb (shown in Table 2). The sequence of DNS-1 was anchored to seven chromosomes (shown in Figure 2). In 458 CEGs, 448 were completely present, indicating that 97.82% of the CEGs were detected. In 248 highly conserved CEGs, 234 were completely present, indicating that 94.35% of the highly conserved CEGs were detected. The results of assessment showed the high completeness of the assembly (shown in Table S6). BUSCO evaluation revealed that the integrity was 85.69% and the percentage of duplicated BUSCO was 3.19% (shown in Table S7 and Figure S4). According to the evaluation results, the genome redundancy sequence was significantly reduced by Hi-C optimization and the integrity and continuity of the genome were of high quality.

**Figure 3:**
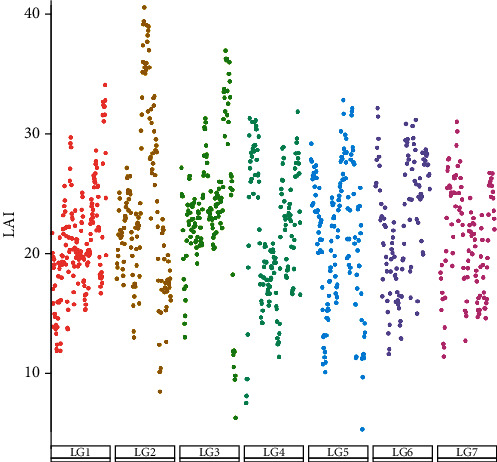
LAI score in DNS-1. LAI score reveals assembly quality in DNS-1. *x*-axis indicates chromosomes of each genome.

The genome quality was classified by LAI, according to genome researchers. For gold quality, the LAI score was greater than 20; for the reference quality, the LAI score was between 10 and 20; and for draft quality, the LAI score was less than 10 [17]. Our data indicated that the LAI score of the DNS-1 genome assembly was 21.32, belonging to gold quality (shown in Figure 3).

### 3.2. Repetitive Sequence

Playing important roles in the evolution of genomes, repetitive sequences were identified [41, 42], using structure-based and homology-based searches. In the DNS-1 genome (321.29 Mb), approximately 52.3% of the assembly sequences were annotated as repetitive sequences (shown in Table S8). This ratio of repetitive elements of red raspberry is higher than that of any other sequenced Rosaceae species such as peach (29.6%) [7], strawberry (22.0%) [2], and apple (43.3%) [1]. And it is similar to black raspberry (56%) [43] and pear (53.1%) [4]. LTR retrotransposons are ubiquitous eukaryotic transposable elements, which play important roles in structure, distribution, and functions among chromosomes [4, 18]. LTR represented 24.15% (77.6 Mb) of all sequences: Gypsy-type retrotransposons and Copia-type retrotransposons occupying 15.54% (49.93 Mb) and 8.61% (27.67 Mb), respectively. Long interspersed elements (LINES) comprised 30,253 elements and PLE|LARD were surprisingly abundant with 99,345 elements accounting for 2.95% and 18.98%, respectively. DNA transposons accounted for 4.64% of the red raspberry genome, lower than peach (9.05%), black raspberry (8.1%), and strawberry (5.16%) and higher than apple (0.9%) and pear (2.41%). The most abundant family in DNA transposons was terminal inverted repeat (TIR) with 22,835 elements occupying 2.57% in the whole genome.

**Figure 4:**
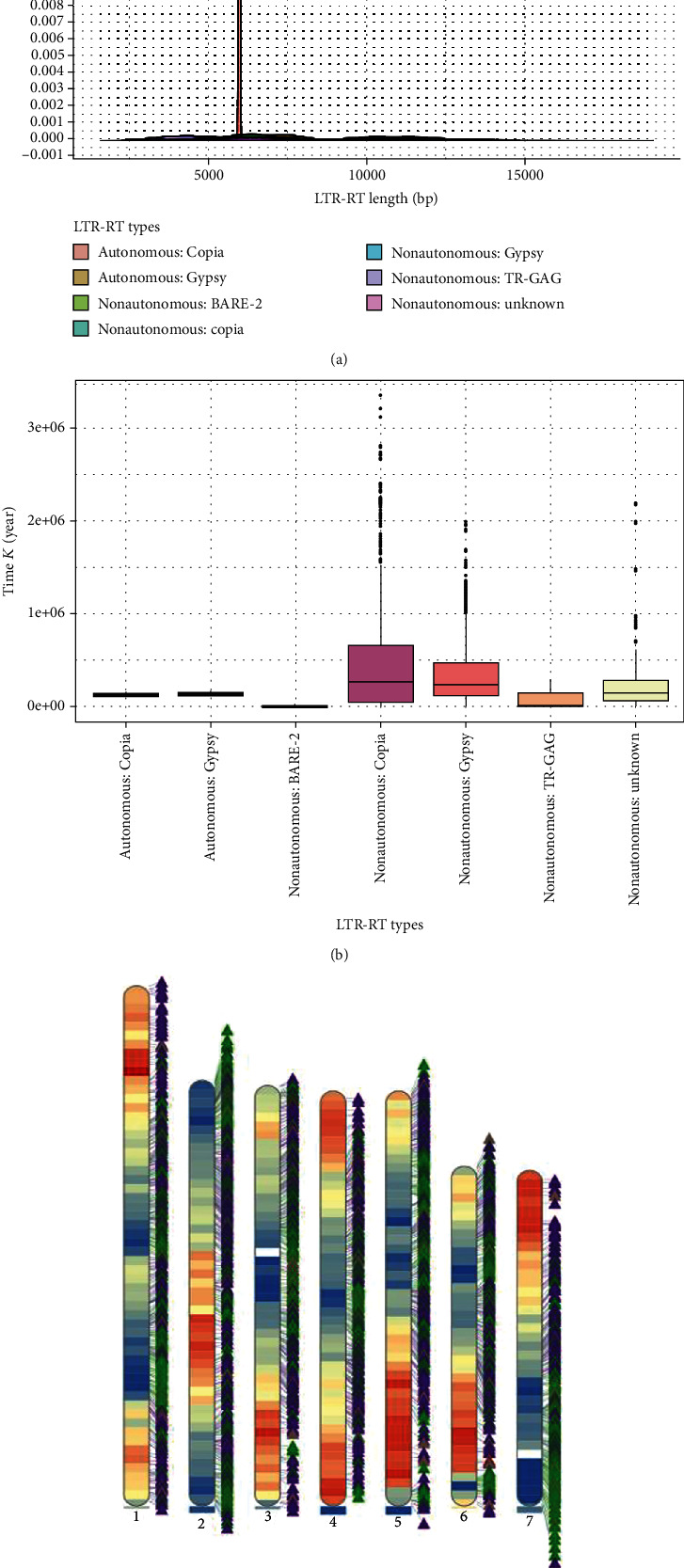
MegaLTR generated results of DNS-1. (a) The distribution of LTR-RT length. (b) Boxplot of insertion age. (c) The visualized density of genes and LTR-RTs on chromosomes.

MegaLTR is a web server and pipeline that detects intact LTR-RTs. Using multiple tools, such as LTR_FINEDR, LTRharvest, and LTR_retriever, it integrates the structure-based, homology-based, and de novo–based intact LTR-RT identification, annotation, and classification [44]. The length of LTR-RT, the visualized density of genes, the age of insertion of LTR-RT, and LTR-RTs on chromosomes were analyzed with the DNS-1 genome (shown in Figure 4).

All intact LTRs were used to predict the estimate insertion ages. Compared with that of other sequenced plant species, including *Arabidopsis thaliana*, *Fragaria vesca*, *Prunus persica*, *Pyrus* × *bretschneideri*, and *Vitis vinifera*, *Rubus idaeus* L. had a high LTR expansion rate (shown in Figure S5). Nevertheless, this result might be affected by variable environmental conditions and the assembly methods.

MegaSSR is an online server that identifies SSR and enables the design of SSR markers at the whole genome [45]. The results were shown in supporting information, including the Venn diagram of identified genic and nongenic SSR (Figure S6A), distribution of the different SSR classes (Figure S6B), SSR distribution considering sequence complementarity (Figure S6C), frequency of the identified SSR motifs (Figure S6D), shared repeats between genic and nongenic regions (Figure S6E), unique repeats in the genic region (Figure S6F), and nongenic region (Figure S6G).

### 3.3. Gene Predictions and Functional Annotations

A total of 29,814 protein-coding genes were predicted to be in DNS-1 with the EVidenceModeler pipeline [30] incorporating homology-based search, ab initio predictions, and RNA-Seq data (shown in Table S9). In predicted 29,814 genes, 97.20% could be annotated including NR, KOG, GO, KEGG, and TrEMBL databases (shown in Table S10). The KOG database is based on the phylogenetic relationships of proteins encoded by bacteria, algae, and eukaryotes with complete genomes. In the KOG analysis, the significant function involved transcription, posttranslational modification, protein turnover, and signal transduction mechanisms (shown in Figure S7). KEGG pathway–based analysis is helpful to further understand the biological functions of genes. In the GO analysis, the genes were assigned to the GO terms of cellular component, molecular function, and biological process, respectively (shown in Figure S8). We also identified 2474 pseudogenes in the DNS-1 genome using the GeneWise v2.4.1 software [31].

### 3.4. Gene Family

For comparing the genes with *Pyrus* × *bretschneideri*, *Solanum lycopersicum*, *Prunus avium*, *Malus* × *domestica*, *Vitis vinifera*, *Arabidopsis thaliana*, and *Prunus persica*, a total of 29,814 genes were observed in *Rubus idaeus* L., and the 24,982 genes were divided into 14,464 gene families predicted in OrthoMCL [36]. Among all the gene families, 652 gene families were unique to *Rubus idaeus* L. (shown in Table S11 and Figure S9).

### 3.5. Phylogenetic Tree Construction and Divergence Time Estimation

**Figure 5:**
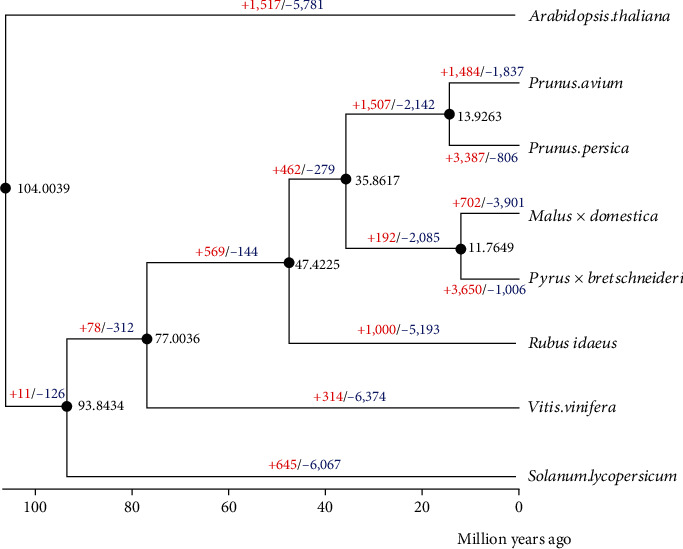
The estimation of divergence time and expansion and contraction of gene families. The expansion (red) and contraction (blue) of gene families and the divergence time (Mya) were estimated in *Rubus idaeus* L., *Solanum lycopersicum*, *Vitis vinifera*, *Arabidopsis thaliana*, *Malus × domestica*, *Prunus persica*, *Prunus avium*, and *Pyrus × bretschneideri*.

Subsequently, comparing single-copy protein sequences of *Rubus idaeus* L. with *Solanum lycopersicum*, *Vitis vinifera*, *Arabidopsis thaliana*, *Malus* × *domestica*, *Prunus persica*, *Prunus avium*, and *Pyrus* × *bretschneideri*, the phylogenetic tree of evolution was constructed by PhyML. *Rubus idaeus* L. had the closest relationship with *Pyrus* × *bretschneideri*, *Malus* × *domestica*, *Prunus persica*, and *Prunus avium*, and they were clustered into one monophyletic group (shown in Figure S10). We then used the MCMCTree method as implemented in the PAML package (v.4.4) to estimate the divergence times of *Rubus idaeus* L. from the other plants. The divergence time of sequenced plant species has been estimated according to known fossil time, and the results are shown in Figure 5. The results showed that the hypothesis of a close relationship was well established among *Rubus idaeus* L., *Pyrus* × *bretschneideri*, *Malus* × *domestica*, *Prunus persica*, and *Prunus avium*. The estimated divergence time of *Rubus idaeus* L., *Pyrus* × *bretschneideri*, *Malus* × *domestica*, *Prunus persica*, and *Prunus avium* was 47.4225 Mya (shown in Figure 5).

### 3.6. WGD and Gene Family Expansion Analysis

**Figure 6:**
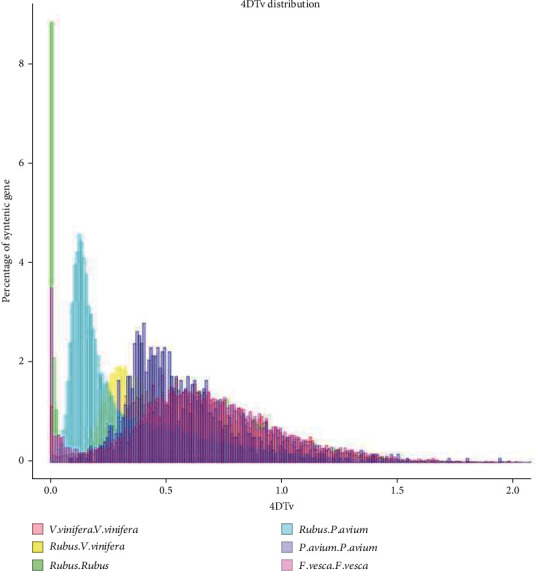
4DTv distribution of whole-genome duplication (WGD). 4DTv analyses among *Vitis vinifera*, *Rubus idaeus* L., *Prunus avium*, and *Fragaria vesca* were estimated. The percentages of the syntenic gene were calculated by 4DTv values, including the paralogous gene pairs within *V. vinifera*, *Rubus*, *F. vesca*, and *P. avium*; the pairs between *Rubus* and *V. vinifera*; and the pairs between *Rubus* and *P. avium*.

To further analyze the WGD events of *Rubus idaeus* L. and other species, we calculated the percentage of transversions at 4DTv distribution of homologous gene mutation. A peak 4DTv value for the orthologs between *Rubus idaeus* L. and *Prunus avium* was 0.13 (shown in Figure 6). According to the results of the evolutionary relationship and the clustering of the gene family, CAFE [39] was used to identify the contraction and expansion of the gene family. There were 1000 and 5193 gene families that expanded and contracted in the *Rubus idaeus* L. genome. The functional annotation analysis of some expansion gene families is shown in Table S12. The expansion gene involved in plant protein of unknown function, FAD-binding domain, and plastocyanin-like domain represented the most abundant groups.

## 4. Discussion

The genome of black raspberry was assembled by VanBuren et al. [43] using eight Illumina paired-end libraries, and the size of the genome was 243 Mb. Here, a high-quality genome of *Rubus idaeus* L. (DNS-1) was assembled by Pacific Biosciences SMRT sequencing and Hi-C sequencing technologies. The schematic workflow for the genome assembly was described by previous studies [46, 47]. Using a PacBio long-read sequencing approach, the total assembly of the red raspberry genome was 485.03 Mb. The contig N50 size of red raspberry was 4.91 Mb which was larger than that of the black raspberry genome with 0.35 Mb. The Hi-C technique, which was a powerful method for the identification of variations, supported that the genome contigs or scaffolds were correctly anchored and oriented (shown in Figure S2). Using Hi-C technology, the red raspberry (DNS-1) genome was optimized, and the final genome size was 321.29 Mb, which was similar to recently published data about 291.7 Mb [48] and 231.21 Mb [49], respectively. And the results were also similar to other Rosaceae plant genomes including peach (224.6 Mb), strawberry (240 Mb), and black raspberry (243 Mb). The genome size of DNS-1 is different from that of the other two researchers, which may be due to the different regions, breeding conditions, and assembly tools.

A large number of repeated sequences indicate that genetic materials are constantly self-replicating and exchanging with each other during the evolution of species [50] and play an important role in human-guided crop improvement [43]. In the DNS-1 genome, approximately 52.3% of the assembly sequences were annotated as repetitive sequences (shown in Table S8). These results suggested that the repeated sequences in red raspberry contribute to human-guided crop improvement.

The whole-genome sequencing of *Rubus idaeus* L. may be utilized in biological research, breeding, and agronomically important characteristics, such as disease resistances, vegetation, and fruit quality. Compared with the sequences of *Pyrus* × *bretschneideri*, *Solanum lycopersicum*, *Prunus avium*, *Malus* × *domestica*, *Vitis vinifera*, *Arabidopsis thaliana*, and *Prunus persica*, we found that 652 gene families (24,982 genes) were unique to *Rubus idaeus* L. These gene families may play important roles in biological evolution [51]. We constructed a phylogenetic tree, and the results showed that they were shaped from an ancestor. Using comparative genomic approaches, the DNS-1 genome increased our insight of knowledge about the genome evolution and comparative genomic research of the Rosaceae.

Scientific research results have confirmed that the various bioactive ingredients and nutrients in red raspberry are beneficial to human health. It has been linked to reduced risk and therapeutic effect for many chronic diseases, including cardiovascular disease, Type 2 diabetes, and different types of tumors [52–57]. Studies have shown that red raspberry and its polyphenols exhibit antitumor effects, and the mechanisms mainly include the elimination of ROS to prevent DNA oxidative damage, enhance DNA repair ability, and regulate cell proliferation, apoptosis, inflammation, angiogenesis, cell cycle arrest, and other signaling pathways. Our previous work indicated that fresh red raspberry extracts had potent abilities to restrain DEN-induced hepatic lesions in rat models. The results suggested that fresh red raspberry extracts could reduce the incidence of hepatocellular adenoma [58]. Our research work also suggested that red raspberry extracts had a potency inhibitory effect on hepatocellular carcinoma cells through modulation of the PTEN/AKT pathway [56]. As a new mechanism in recent years, cross-species regulation of plant microRNA (miRNA) also plays an important role in the regulation of human health and diseases [59–61]. We will pay more attention to the role of cross-species regulation of red raspberry miRNA in human in the future.

## Figures and Tables

**Table 1:** The statistics for the genome of DNS-1.

	**DNS-1**
Contig number	255
Contig length (bp)	485,026,566
Contig N50 (bp)	4,914,301
Contig N90 (bp)	1,040,369
Contig max (bp)	19,804,964
GC content (%)	37.91
Gap total length (bp)	0

**Table 2:** The Hi-C statistics of the DNS-1 genome.

	**DNS-1**
Contig number	451
Contig length (bp)	321,297,455
Contig N50 (bp)	2,360,341
Contig N90 (bp)	240,636
Contig max (bp)	14,017,073
GC content (%)	37.96
Gap total length (bp)	0

## Data Availability

The data that support the findings of this study are openly available in the National Center for Biotechnology Information. The raw sequencing data and Hi-C data of the genome were deposited in the genome database of the National Center for Biotechnology Information under BioProject ID PRJNA515339, and the BioSample accession numbers are SAMN10754102 and SAMN10744216, respectively.

## Supporting Information

Supporting Information Additional supporting information can be found online in the Supporting Information section. Table S1. Distribution of subread length. Table S2. Completeness analysis of the *Rubus idaeus* L. genome based on the CEG database. Table S3. The results of BUSCO evaluation. Table S4. Each type of Hi-C sequencing data. Table S5. The assemblies of Hi-C data. Table S6. Completeness analysis of Hi-C data based on the CEG database. Table S7. BUSCO evaluation of Hi-C data. Table S8. Repetitive sequences in *Rubus idaeus* L. Table S9. The results of gene prediction. Table S10. Gene function annotation with different databases. Table S11. Genetic family. Table S12. Gene function annotation of expansion gene families. Figure S1. The distribution curve of *k*-mers frequency (*k* − mers = 19) in the *Rubus idaeus* L. genome. Figure S2. The figure of BUSCO assessment results. Figure S3. Hi-C intrachromosomal links of ordered scaffolds for each chromosome. Figure S4. BUSCO assessment results of Hi-C data. Figure S5. The distribution of LTR insertion time. Figure S6. Detection of SSRs at the whole-genome level. Figure S7. KOG functional classification of annotated genes. Figure S8. GO categories of the annotated genes. Figure S9. Gene families. Figure S10. Phylogenetic tree across *Solanum lycopersicum*, *Vitis vinifera*, *Rubus idaeus* L., *Pyrus* × *bretschneideri*, *Malus* × *domestica*, *Prunus persica*, *Prunus avium*, and *Arabidopsis thaliana*.
