# Supplementary material for: The High-Quality Genome Sequencing and Analysis of Red Raspberry (Rubus idaeus L.)
Source: Int J Genomics. 2024 Nov 25;2024:9271183. doi: 10.1155/2024/9271183 (PMC11611404; doi:10.1155/2024/9271183)
Supplement: Supporting Information — Additional supporting information can be found online in the Supporting Information section. Table S1. Distribution of subread length. Table S2. Completeness analysis of the Rubus idaeus L. genome based on the CEG database. Table S3. The results of BUSCO evaluation. Table S4. Each type of Hi-C sequencing data. Table S5. The assemblies of Hi-C data. Table S6. Completeness analysis of Hi-C data based on the CEG database. Table S7. BUSCO evaluation of Hi-C data. Table S8. Repetitive sequences in Rubus idaeus L. Table S9. The results of gene prediction. Table S10. Gene function annotation with different databases. Table S11. Genetic family. Table S12. Gene function annotation of expansion gene families. Figure S1. The distribution curve of k-mers frequency (k − mers = 19) in the Rubus idaeus L. genome. Figure S2. The figure of BUSCO assessment results. Figure S3. Hi-C intrachromosomal links of ordered scaffolds for each chromosome. Figure S4. BUSCO assessment results of Hi-C data. Figure S5. The distribution of LTR insertion time. Figure S6. Detection of SSRs at the whole-genome level. Figure S7. KOG functional classification of annotated genes. Figure S8. GO categories of the annotated genes. Figure S9. Gene families. Figure S10. Phylogenetic tree across Solanum lycopersicum, Vitis vinifera, Rubus idaeus L., Pyrus × bretschneideri, Malus × domestica, Prunus persica, Prunus avium, and Arabidopsis thaliana. [file 9271183.f1.docx]

**Figure S1. The distribution curve of *k*-mers frequency (*k*-mers = 19) in *Rubus idaeus* L. genome.**


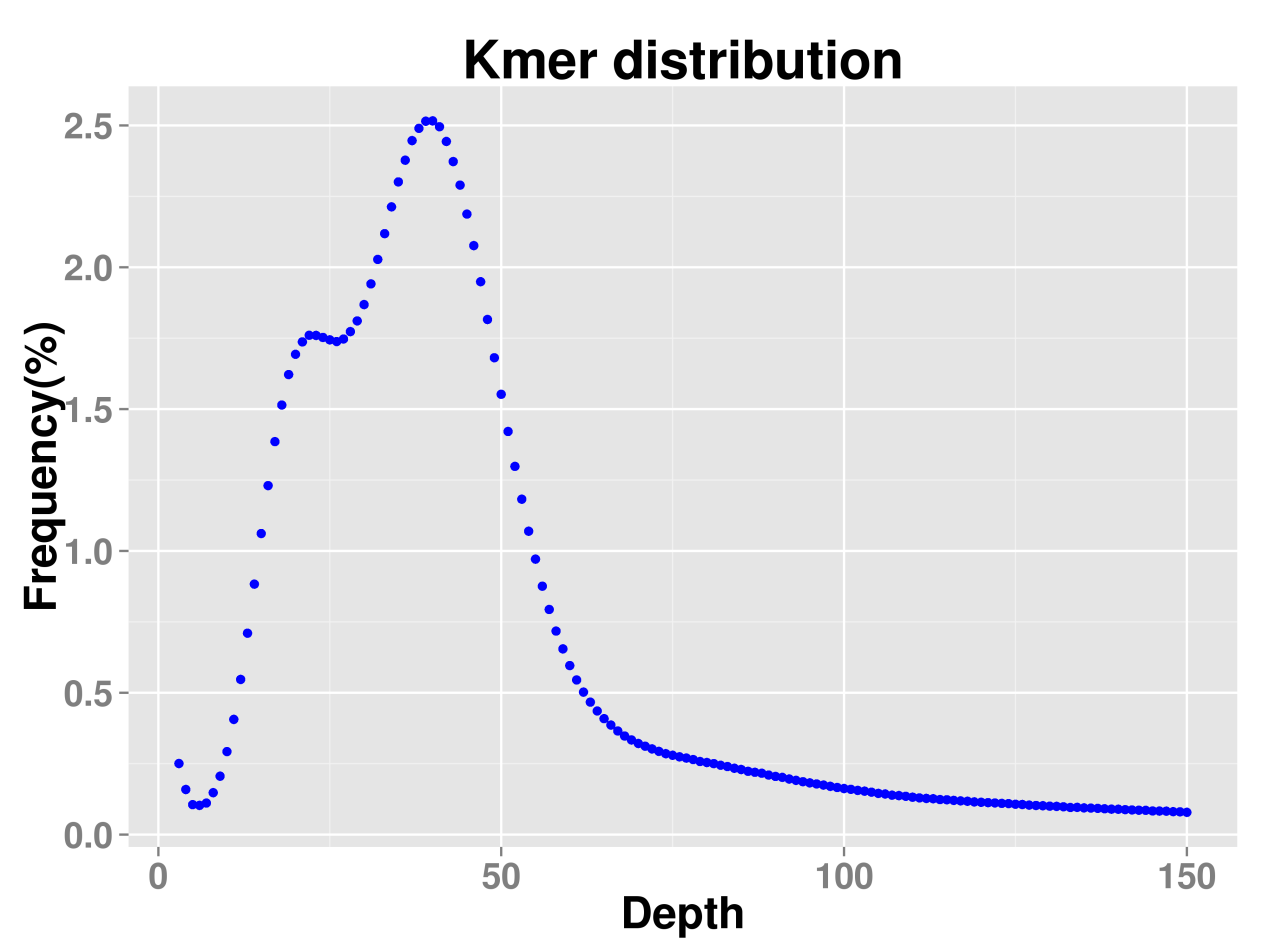


**Figure S2. The figure of BUSCO assessment results.**


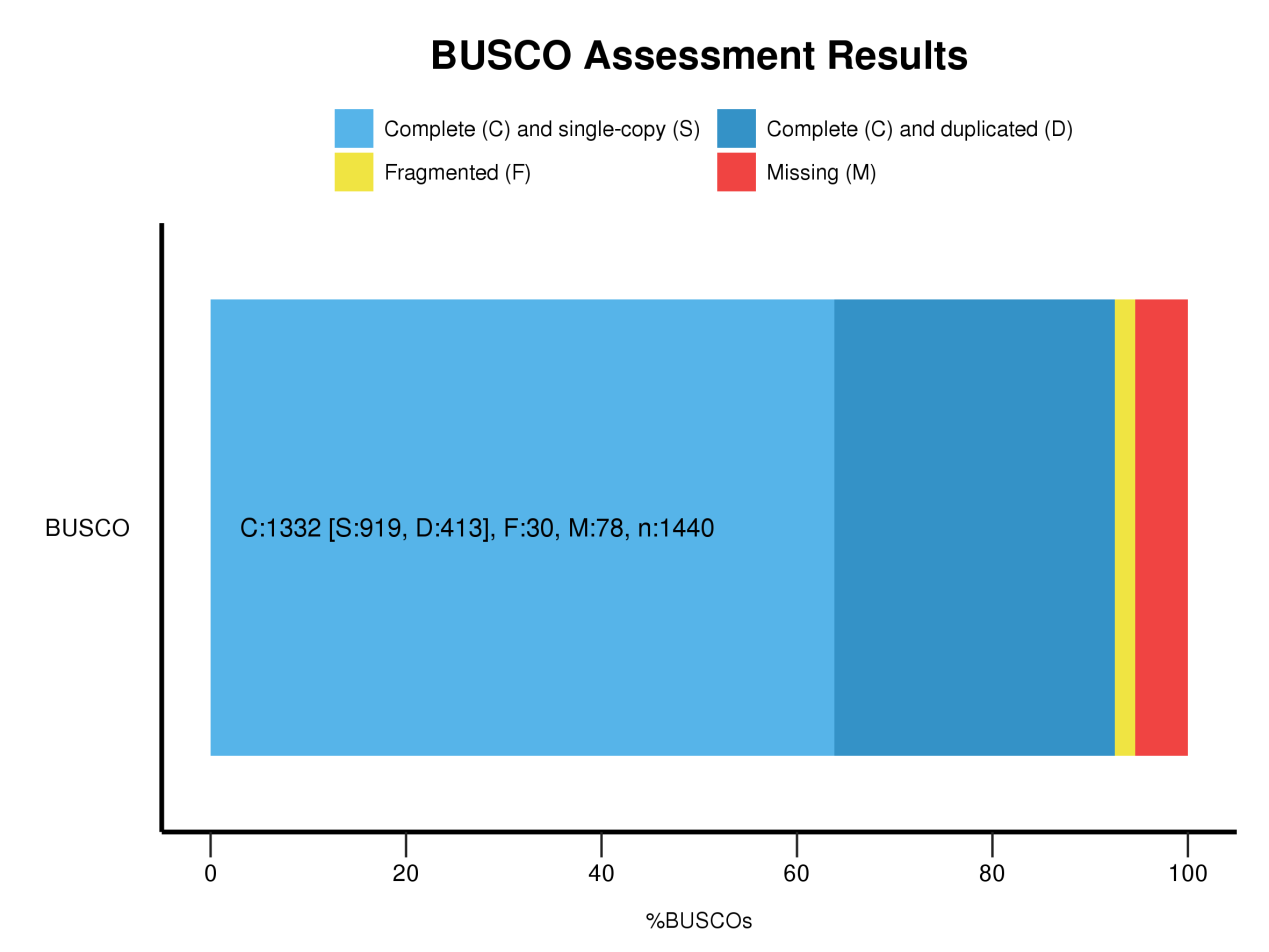


**Figure S3. Hi-C intra-chromosomal links of ordered scaffolds for each chromosome.**

**
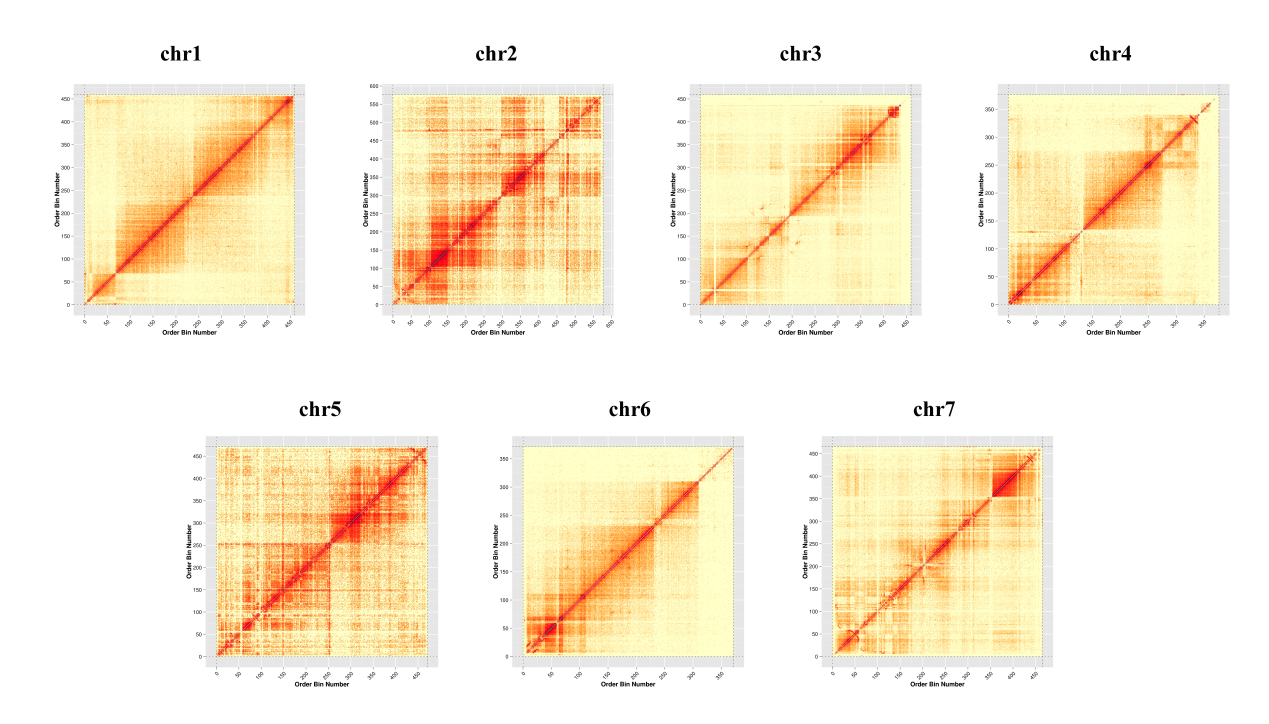
**

**Figure S4. BUSCO assessment results of Hi-C data.**


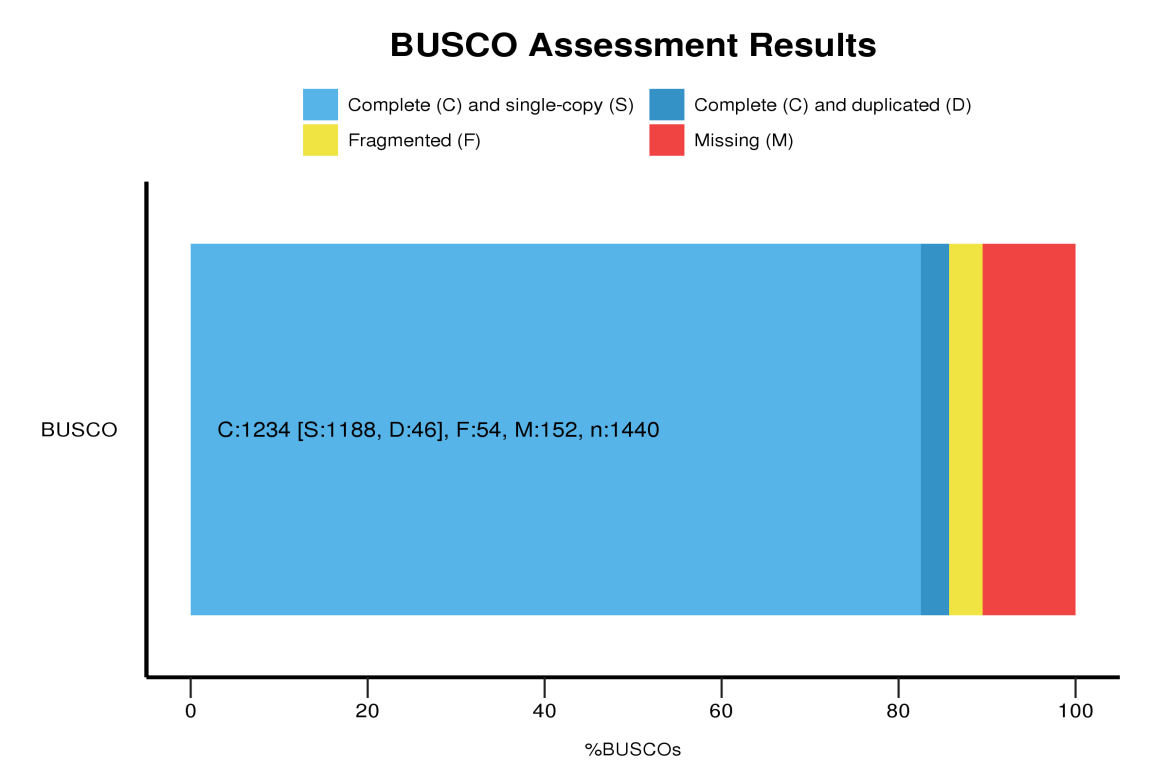


**Figure S5. The distribution of LTR insertion time.**


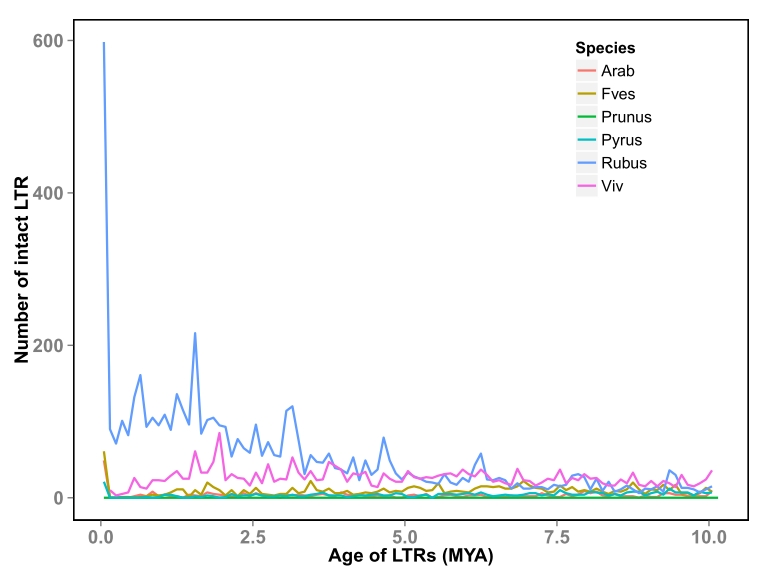


**Figure S6. Detection of SSRs at the whole genome level.**

**
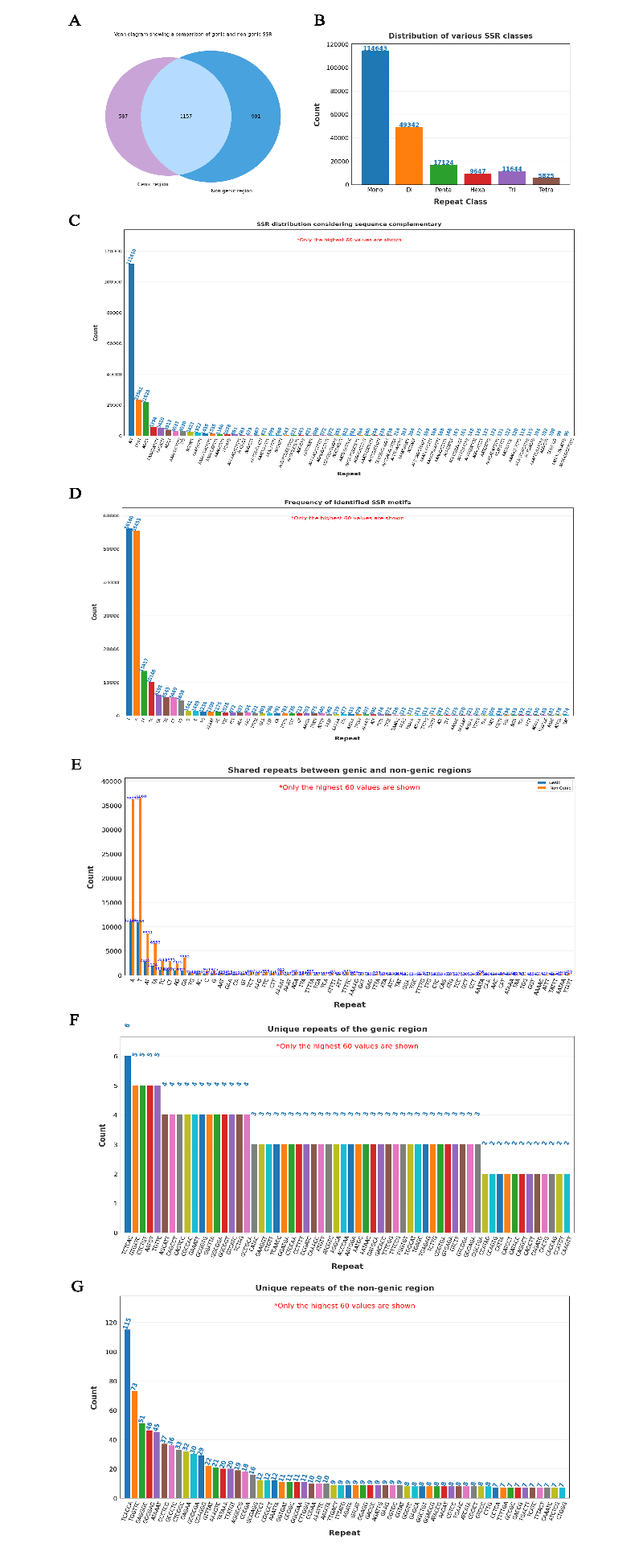
**

**Figure S7. KOG functional classification of annotated genes.**


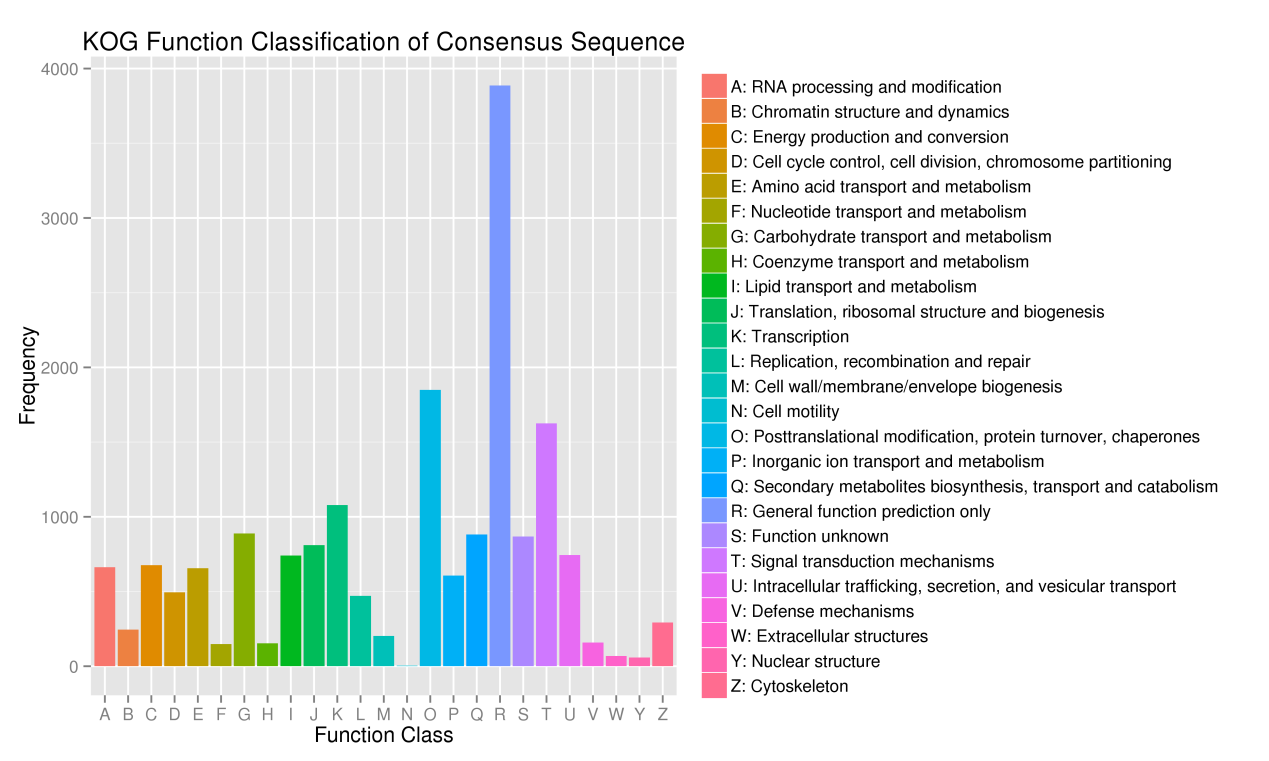


**Figure S8. GO categories of the annotated genes.**


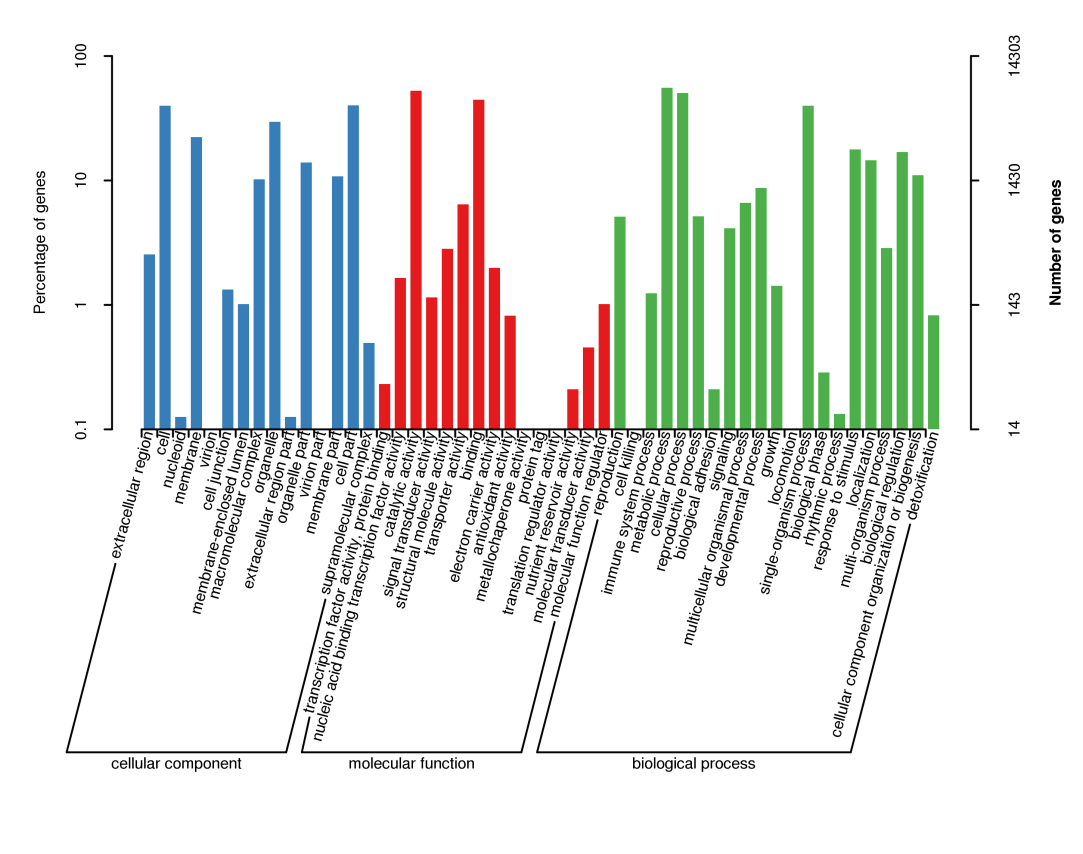


**Figure S9. Gene families.**


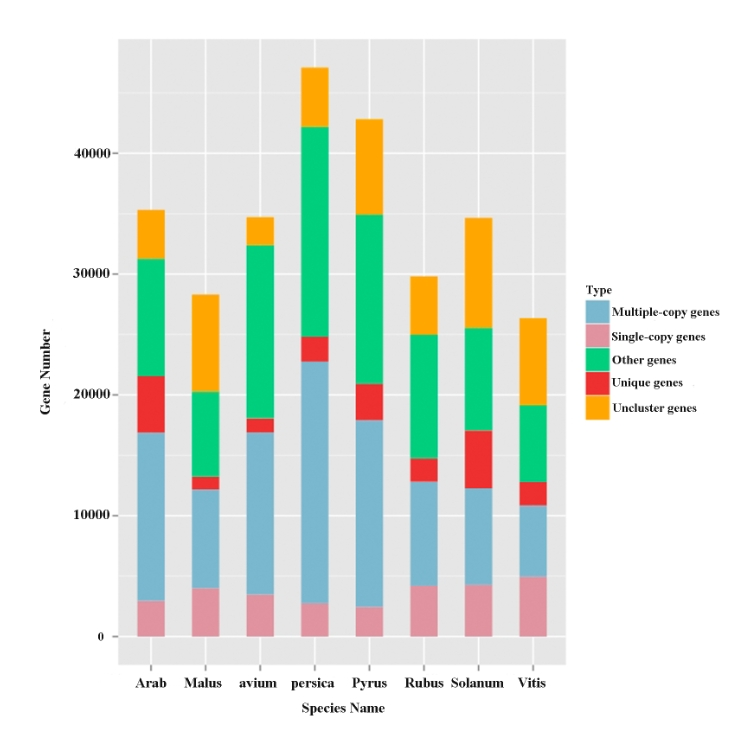


**Figure S10. Phylogenetic tree across *Solanum.lycopersicum*, *Vitis.vinifera*, *Rubus idaeus* L., *Pyrus.x.bretschneideri*, *Malus.x.domestica*, *Prunus.persica*, *Prunus.avium*., and *Arabidopsis.thatliana*.**


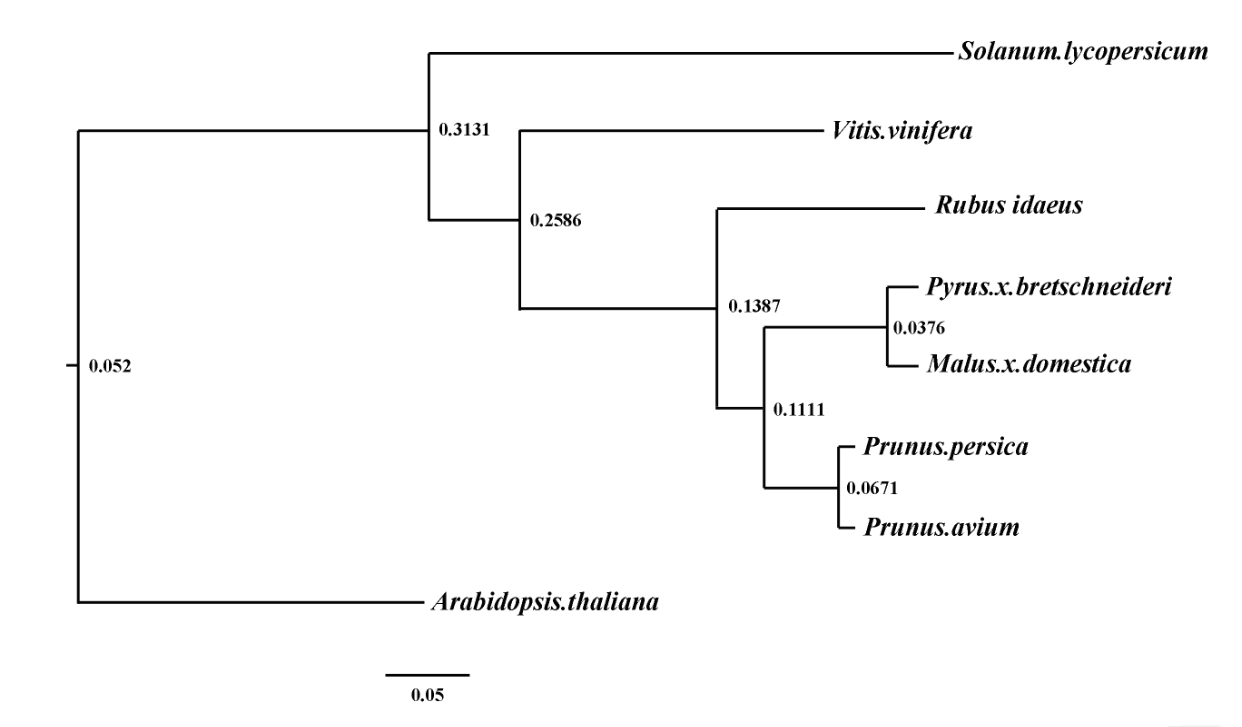


**Table S1. Distribution of subreads length.**

| Length (bp) | Number | Total length (bp) | Average length (bp) |
| --- | --- | --- | --- |
| 500~2,000 | 196,404 | 237,992,960 | 1,211.75 |
| 2,000~4,000 | 202,818 | 598,594,529 | 2,951.39 |
| 4,000~6,000 | 160,286 | 796,851,810 | 4,971.44 |
| 6,000~8,000 | 136,967 | 955,983,858 | 6,979.67 |
| 8,000~10,000 | 122,690 | 1,102,505,534 | 8,986.11 |
| 10,000~12,000 | 123,202 | 1,356,708,797 | 11,012.07 |
| 12,000~14,000 | 130,832 | 1,700,259,162 | 12,995.74 |
| 14,000~16,000 | 119,615 | 1,791,221,277 | 14,974.89 |
| 16,000~18,000 | 100,152 | 1,699,530,596 | 16,969.51 |
| 18,000~ | 546,451 | 16,192,372,557 | 29,631.88 |
| Total | 1,839,417 | 26,432,021,080 | 14,370 |

**Table S2. Completeness analysis of *Rubus idaeus* L. genome based on CEG database.**

| Species | Number of 458  CEGs present in  assembly | Percent of 458 CEGs  present in  assemblies | Number of 248  highly conserved  CEGs present | Percent of 248 highly  conserved CEGs  present |
| --- | --- | --- | --- | --- |
| *Rubus idaeus* L. | 440 | 96.06 % | 235 | 94.76 % |

**Table S3. The results of BUSCO evaluate**

| Species | Complete  BUSCOs | Complete and single-copy  BUSCOs | Complete and duplicated  BUSCOs | Fragmented  BUSCOs | Missing  BUSCOs |
| --- | --- | --- | --- | --- | --- |
| *Rubus idaeus* L. | 1,332 | 919 | 413 | 30 | 78 |

**Table S4. Each type of Hi-C sequencing data.**

| Type | Number | Ratio(%) |
| --- | --- | --- |
| Unique Paired Alignments | 27,335,043 | 100 |
| Valid Interaction Pairs | 21,933,221 | 80.24 |
| Dangling End Pairs | 1,413,512 | 5.17 |
| Re-ligation Pairs | 432,003 | 1.58 |
| Self-cycle Pairs | 2,306,016 | 8.44 |
| Dumped Pairs | 1,250,291 | 4.57 |

**Table S5. The assembles of Hi-C data.**

| Group | Sequence  Number | Sequence Length(bp) |
| --- | --- | --- |
| Lachesis Group 1 | 56 | 88,542,335 |
| Lachesis Group 2 | 149 | 73,870,309 |
| Lachesis Group 3 | 84 | 75,592,044 |
| Lachesis Group 4 | 58 | 65,535,807 |
| Lachesis Group 5 | 109 | 65,100,553 |
| Lachesis Group 6 | 47 | 58,339,537 |
| Lachesis Group 7 | 76 | 55,454,163 |
| Total Sequences Clustered (Ratio %) | 579(96.82) | 482,434,748(99.47) |
| Total Sequences Ordered and Oriented (Rotio %) | 412(71.16) | 318,654,525(66.05) |

**Table S6. Completeness analysis of Hi-C data based on CEG database.**

| Species | Number of 458  CEGs present in  assembly | Percent of 458 CEGs  present in  assemblies | Number of 248  highly conserved  CEGs present | Percent of 248 highly  conserved CEGs  present |
| --- | --- | --- | --- | --- |
| *Rubus idaeus* L. | 448 | 97.82 % | 234 | 94.35 % |

**Table S7. BUSCO evaluation of Hi-C data.**

| Species | Complete  BUSCOs | Complete and single-copy  BUSCOs | Complete and duplicated  BUSCOs | Fragmented  BUSCOs | Missing  BUSCOs |
| --- | --- | --- | --- | --- | --- |
| *Rubus idaeus* L. | 1,234 | 1,188 | 46 | 54 | 152 |

**Table S8. Repetitive sequences in *Rubus idaeus* L..**

| Type | | Number | Length(bp) | Percentage of  assembly(%) |
| --- | --- | --- | --- | --- |
| ClassI/DIRS | 5,956 | 4,400,434 | 1.37 |  |
| ClassI/LINE | 30,253 | 9,473,533 | 2.95 |  |
| ClassI/LTR | 636 | 838,176 | 0.26 |  |
| ClassI/LTR/Copia | 40,756 | 27,670,417 | 8.61 |  |
| ClassI/LTR/Gypsy | 53,188 | 49,934,896 | 15.54 |  |
| ClassI/PLE\|LARD | 99,345 | 60,997,795 | 18.98 |  |
| ClassI/SINE | 1,853 | 398,923 | 0.12 |  |
| ClassI/TRIM | 1,330 | 814,778 | 0.25 |  |
| ClassI/Unknown | 231 | 77,790 | 0.02 |  |
| ClassII/Helitron | 13,284 | 4,961,314 | 1.54 |  |
| ClassII/MITE | 3,356 | 804,927 | 0.25 |  |
| ClassII/Maverick | 586 | 179,058 | 0.06 |  |
| ClassII/TIR | 22,835 | 8,255,293 | 2.57 |  |
| ClassII/Unknown | 3,884 | 699,414 | 0.22 |  |
| PotentialHostGene | 17,509 | 8,766,893 | 2.73 |  |
| Unknown | 60,655 | 18,749,532 | 5.83 |  |
| Total with overlap | 356,102 | 197,215,384 | 61.37 |  |
| Total without overlap | 356,102 | 168,086,380 | 52.31 |  |

**Table S9. The results of gene prediction.**

| Method | | Software | Species | Gene number |
| --- | --- | --- | --- | --- |
|  | Genscan | - | 22,111 |  |
|  | Augustus | - | 35,119 |  |
| Ab initio | GlimmerHMM | - | 31,796 |  |
|  | GeneID | - | 33,975 |  |
|  | SNAP | - | 48,487 |  |
|  |  | *Arabidopsis_thaliana* | 23,919 |  |
|  |  | *Fragaria_vesca* | 26,706 |  |
| Homology-based | GeMoMa | *Fragaria iinumae* | 49,556 |  |
|  |  | *Fragaria nipponica* | 62,132 |  |
|  |  | *Fragaria nubicola* | 62,456 |  |
| RNAseq | PASA | - | 28,855 |  |
| Integration | EVM | - | 29,814 |  |

**Table S10. Gene function annotation with different database.**

| Database | Annotated number | Percentage(%) |
| --- | --- | --- |
| GO_Annotation | 14,303 | 47.97% |
| KEGG_Annotation | 10,045 | 33.69% |
| KOG_Annotation | 16,177 | 54.26% |
| TrEMBL_Annotation | 28,701 | 96.27% |
| NR_Annotation | 28,958 | 97.13% |
| All_Annotated | 28,978 | 97.20% |

**Table S11. Genetic family.**

| Species name | Total gene  number | Cluster gene number | Total family number | Unique gene family number |
| --- | --- | --- | --- | --- |
| *Rubus idaeus* | 29,814 | 24,982 | 14,464 | 652 |
| *Pyrus.x.bretschneideri* | 42,811 | 34,913 | 15,871 | 1,199 |
| *Solanum.lycopersicum* | 34,651 | 25,568 | 13,643 | 1,136 |
| *Prunus.avium* | 34,705 | 32,420 | 15,578 | 363 |
| *Malus.x.domestica* | 28,306 | 20,278 | 12,613 | 421 |
| *Vitis.vinifera* | 26,346 | 19,168 | 12,608 | 683 |
| *Arabidopsis.thaliana* | 35,313 | 31,281 | 13,246 | 1,339 |
| *Prunus.persica* | 47,089 | 42,212 | 16,476 | 707 |

**Table S12. Gene function annotation of expansion gene families.**

| **Gene family** | **Pfam** | **Function** |
| --- | --- | --- |
| GF_8779 | PF05687.8 | Plant protein of unknown function (DUF822) |
| GF_1497 | PF00667.15 | FAD binding domain |
| GF_13684 | PF02298.12 | Plastocyanin-like domain |
| GF_16625 | PF00565.12 | Staphylococcal nuclease homologue |
| GF_2119 | PF00696.23 | Amino acid kinase family |
| GF_10547 | PF08238.7 | Sel1 repeat |
| GF_1469 | PF04616.9 | Glycosyl hydrolases family 43 |
| GF_2616 | PF03572.13 | Peptidase family S41 |
| GF_6028 | PF03105.14 | SPX domain |
| GF_2028 | PF03062.14 | MBOAT, membrane-bound O-acyltransferase family |
